# *There is no item* vs. *I wish there were an item*: Implicit negation causes false recall just as well as explicit negation

**DOI:** 10.1371/journal.pone.0215283

**Published:** 2019-04-12

**Authors:** Józef Maciuszek, Mateusz Polak, Martyna Sekulak

**Affiliations:** Institute of Applied Psychology, Faculty of Management and Social Communication, Jagiellonian University, Cracow, Poland; University of St Andrews, UNITED KINGDOM

## Abstract

When talking about absence, we may express it in a negative statement (using explicit negation e.g. *I was not*) or in a positive statement (using implicit negation e.g. *I wished I were*). Previous research has shown that explicitly negated statements may cause false recall–negated items may paradoxically be remembered as present. The current study compares false recall caused by implicit and explicit negation. Participants listened to a recording in which some objects were mentioned as present, some as absent, and some not mentioned at all. The absence of objects was expressed using explicit or implicit negation. Participants’ recall of the recording was measured either five minutes or one week after exposure to the recording. Results indicate that implicit and explicit negation lead to a nearly identical false recall of negated items. However, items not mentioned in the recording (i.e. neither mentioned nor negated) were more often recognized as present by participants exposed to implicit, rather than explicit negation. We postulate that false recall of negated items could be explained by participants remembering the item itself, but forgetting the context in which it was present (an affirmative or a negative statement), hence objects would be recalled as present just because they were spoken of.

## Introduction

In everyday communication, one of the main functions of negation is to inform about the absence of objects (e.g. There *is no fireplace in the living room*), as well as to deny events (e.g. *Yesterday*, *the last flight from Washington was not late*) and behavior (e.g. *John did not stop at a red light*). How negation influences the memory of negated objects or behavior is of interest both from a scientific (e.g. memory and language theories) and practical standpoint (e.g. forensic psychology). Many psycholinguistic studies have found that negatives are harder to process than affirmatives [1, 2, 3] Negative statements impede memory (e.g. [4]) and can have unintended, paradoxical effects for the recipients of the communication [5, 6, 7]. Results of many previous studies (e.g. [4, 8, 9]) showed that people remember affirmative sentences better than negative ones. The research question investigated in a paper by Maciuszek & Polczyk [10] was whether recalling negated objects and actions can have adverse effects on memory, namely result in a higher rate of false recognition as compared to when the items are not mentioned at all. The aforementioned study [10] demonstrated that after a one-week delay, negated items (i.e. items that the source material stated were not present) were more often reported as ‘Present’ than items not mentioned at all. These results were similar when objects and actions were negated.

The present research aims to test whether the aforementioned higher rate of false recall is caused directly by the cognitive processing of an explicitly negative sentence (i.e. using the words *no*, *not*, *absent*, etc.), or is it possible to cause said false memories using other grammatical constructs to convey negation.

In order to investigate the above question, we decided to use implicit negation to inform participants about the absence of objects within affirmative sentences. With implicit negation, objects or actions are not directly denied, but rather their absence needs to be inferred from the statement (i.e. it is an implication of the sentence–as a presupposition or implicature, rather than the sentence’s entailment). For example, such implicitly negative statements may describe causes or consequences of the object or behavior not being present, without directly stating its absence. It should be noted that there are very few studies on implicit negation in psychology or linguistics, and we failed to find any research on it in the context of memory. This paper compares the memory effects of explicit and implicit negation. We investigate whether the false recall of negated items is to be explained by the cognitive processing of explicitly negative statements using the words *no*, *not*, *none*, *absent*, etc., or are other explanations required.

### Previous studies on memory of negated *versus* not mentioned objects

In a previous study [10] it was tested whether negating the presence of an object or behavior may lead to incorrect recall that the negated object was actually present or that the negated behavior actually did occur. The authors call this effect “negation related false memories” (NRFM). The presence of NRFM was tested by exposing participants to an audio description of a newly-bought house (or in another experiment—an audio description of the actions taken by a person driving a car) in which some objects (or actions) were mentioned, some were negated, and others were not mentioned at all (i.e. did not appear in the description). Thus, the materials to be remembered in Experiments 1 and 2 of the aforementioned study pertained to a static description of a house, whereas the materials to be remembered in Experiment 3 were related to human behavior—driving a car (either following or breaking various rules of the Highway Code by the driver). A memory recognition test was performed either five minutes after the presentation of the description or one week later. In the short-delay condition (five minutes) the difference in recall between items which were negated and not mentioned was not significant. After a week, negated items (i.e. items which the source stated were absent) were recognized as present more often than items which were not mentioned in the source at all. This effect was replicated in all three experiments. Moreover, in experiment 2 and 3 the difference in the number of false recognitions of negated items was not statistically different from the number of correct recognitions of items described as present in the source.

The authors explained the results by referring to the cognitive processing of negative statements, such as the increased cognitive load and difficulty of processing negation, the dissociation of the negation tag, and paradoxical activation of mental representations of the negated ideas. They assumed that results should be discussed in terms of the retention hypothesis [11] or based on the two-step simulation hypothesis of negation [12].

The idea behind the present study was to investigate whether the NRFM effect is associated with a different memory mechanism than processing explicit negation. For example, one may assume that it is easier to remember the name or label of the object present in a statement than the syntax of the sentence (negative or affirmative). It is natural that the information that something was missing or absent usually appears in negated sentences (e.g. *There was no fireplace in the house*). Absence of objects can however be stated using affirmative sentences (e.g. *I wish there were a fireplace in the house*). This is a case of implicit negation. The research question was whether such implicit negation has a different impact on memory than explicitly negated sentences.

### Implicit and explicit negation

There are many different ways to express negation (see [13]). Clark [14] presented the general distinction between asserted and non-asserted negation. The first type is called *explicit* negation and the second—*implicit* negation (see [15]).

It is clear that *no*, *not*, *never* etc. constitute explicit negation. It also includes (in English) such expressions as *scarcely*, *hardly*, *few*, *seldom*, *little*, *only* (see [15]). Implicit negation is conveyed by words such as *forget*, *fail*, *doubt* [15], see also [16]) and is constituted by pragmatic inferences. As noted by Xiang et al. [15] this distinction between the two types of negation is based on the source of the negative meaning: *If negation is expressed as part of the asserted meaning of an utterance*, *i*.*e*., *if it is an entailment*, *it is explicit negation; if it belongs to the non-asserted meaning (i*.*e*., *presupposition or implicature)*, *it is implicit negation*. (p. 72). For example, predicates such as *be surprised*, *be disappointed* trigger negative inferences. When one says that they are surprised, we know that something does not conform to their expectations. The utterance *I am disappointed* means that a certain fact is not in coherence with one’s hopes. *I am disappointed by the quality of the item* implicitly negates the item’s (good) quality. While these examples use emotive predicates to convey implicit negation, it can be expressed in other ways (presented later in this paper).

It should be noted that existing studies on the processing of negation have focused on explicit negation, especially sentential negation (using the word *not*) and as noted by Xiang et al. ([15], p. 72), we know relatively little about how other types of negation are processed.

There are few studies on the processing of implicit negation. It is known that the processing of sentences containing negation is more difficult than of affirmative sentences, regardless of whether the negatives are explicit (e.g. *not*) or implicit (e.g. *forget*) [17, 18]. Sentences become increasingly difficult to process with each instance of (explicit or implicit) negation they contain, potentially causing cognitive overload [19]. There are studies on the role of implicit and explicit negation in the conditional reasoning bias [20]. In their study on the influences of negation and situational presence on the accessibility of text information, Kaup and Zwaan [21] used explicit and implicit negation to inform about the absence of a certain color. The accessibility of the color term was measured by means of a probe-recognition task after the end of the sentence either with a 500 ms or 1500 ms delay. One result which we find especially relevant to our study is that the accessibility of the word-probes (color terms) was similar for implicit and explicit negation (i.e. affirmative absent vs. negative absent). Recent research also includes a study on comprehension of implicit negation measured using ERP (event related potential) [15]. Negative inference (implicit negation) in this study was triggered by “emotive” predicates, e.g. *be amazed*, *be surprised*, *be disappointed*. To sum up, Xiang et al. [15] define implicit negation as presupposed or conventionally implicated–it is non-asserted and is not a sentence’s entailment. Kaup et al. [12] describe affirmative sentences which inform about the absence of objects as *affirmative absents* or *implicit negatives*. Following in their footsteps we define implicit negation as conveyed through the non-asserted, pragmatic negative meaning–inferences beyond entailments, especially as presuppositions and implicatures of the sentences [15].

### Present study

The main problem to be investigated within the presented research was whether the NRFM effect (a significantly higher number of falsely recalled negated items than falsely recalled not mentioned items) after a one-week delay is induced strictly by explicit negation, or whether implicit negation may lead to the same effect, and therefore whether cognitive processing of explicit negation is an adequate explanation of this effect.

It is possible that implicit negation requires a deeper level of cognitive processing as compared to explicit negation–which facilitates better retention [22]. Understanding a sentence such as *I wish there were a fireplace in the house* (implicit negation) not only requires deeper processing than *there is no fireplace in the house* (explicit negation), but may also create better contextual cues by referring to the narrator, their thoughts and emotions, and facilitate encoding specificity [23]. Should this be the case, implicit negation would lead to a lower number of negated items falsely recalled as ‘present’.

The more complex nature of sentences containing implicit negation may, however, cause additional cognitive load required to understand their meaning. As the source used in the presented and previous research concerning NRFM was auditory and therefore time-sensitive, the participants may not have had sufficient cognitive capabilities left to fully process and remember all of the sentences. This would lead to an increased number of negated items falsely recalled as ‘present’ when compared to explicit negation, as well as an increased number of falsely recalled ‘not mentioned’ items (due to guessing and confabulation, as participants would be aware that they did not remember all of the details).

A combination of these effects (deeper cognitive processing and increased load) may lead to a lack of difference in the number of falsely recalled explicitly and implicitly negated items, with an increased number of not mentioned items in the implicit negation treatment.

It is also possible that implicit and explicit negation do not vary in either the depth of cognitive processing or cognitive load, which would lead to a lack of differences between the explicit and implicit negation treatments. Kaup et al. [21] found some similarities in cognitive load between implicit and explicit negation, making this a possible hypothesis. However, their research did not investigate memory or recall, so no strong assumptions about NRFM can be drawn from it.

As one of our main hypotheses concerns a lack of differences between treatments, the insufficient standard NHST statistical tests will be supplemented by calculating Bayesian probabilities of the null and alternative hypotheses for the no-difference effect, as presented by Masson [24].

## Materials and methods

### Participants

One-hundred and eighty psychology students voluntarily took part in the experiment. Fourteen participants were excluded from the analyses because they did not attend the second part of the procedure (eight cases) or filled out the answer sheet incorrectly (four cases). The final sample consisted of 168 participants (139 female, 28 male, one person did not indicate their gender; *M*_age_ = 21.61 years, *SD* = 2.17). Jagiellonian University Institute of Applied Psychology Ethics Committee approved the research on human participants presented in this manuscript. Participation was voluntary and verbal consent was obtained prior to the experimental session. Participants did not receive remuneration.

### Materials

#### Audio recording

We used an audio recording in which the narrator talks about their newly bought house. The description included objects present in the house, as well as items which were absent. The absence of objects was presented in two different ways–in the Explicit Negation version, the narrator used explicit negation to inform about the absence of items (e.g. ‘There are no chairs in the house’), while in the Implicit Negation treatment absence of items was expressed using implicit negation. We used two main types of sentences to convey implicit negation: (1) prospective action to be undertaken by the protagonist (e.g. *I plan to add a fireplace* or *There are windows but I need to buy curtains*) and (2) expressions of disparity between the current and preferred state (e.g. *However*, *I would prefer if there were a playroom* or *I expected there to be a shower*). Implicit negation was derived as an implicature of the statements, based on their context. We purposefully avoided strong emotive predicates (such as *I was furious I have to install the shower myself*), in order to avoid adding emotional recall cues to implicit negation statements while their explicit counterparts are not emotionally loaded. Below are the particular sentences we used to convey implicit negation (implicitly negated [objects] were counterbalanced, and [rooms] changed to reflect everyday language, e.g. not having a [shower] installed in a [kitchen]): (1) *However*, *I expected there to be [object]*, (2) *The [room] would look better if there were [object]*, (3) *The house has [mentioned object] and in the future I am going to have [object] built/added*, (4) *I still need to buy [object] to my [room]*, (5) *I still need to have [object] installed*, and (6) *It would be better if there were a(an) [object]*. In each variant of the recording, there were six items the presence of which was mentioned, six negated items and six items which were not mentioned at all, neither as present nor absent (but were mentioned in other versions of the recording). For counterbalancing purposes, we created twelve versions of the description, so that each item could appear in a different condition (present vs. absent vs. not mentioned) and in both types of negation (explicit vs. implicit). The recordings consisted of 164–174 words and lasted around 2 minutes ± 8 seconds. Due to longer wording required for implicit negation, the Implicit Negation recordings were on average 5 seconds (or 2,5%) longer than Explicit Negation. Word count was identical (average of 170 vs 171 words for Implicit and Explicit Negation, respectively).

#### Memory test

The participants were given a memory recognition test based on the content of the audio recording. It was a paper-and-pencil task in which the participants were asked to match each of the 18 listed items to one of two categories: *Present* or *Not present* in the recording describing the house. Participants were instructed to only mark an object as ‘Present’ if it was explicitly stated in the recording. Depending on the version of the recording, each item might have appeared as present, negated (explicitly or implicitly) or not mentioned. However, in each case six items from the list were present in the building and 12 object were not present. The absence of six of these items was mentioned in the description, while the remaining six objects were not mentioned at all. The memory test measured the number of items classified as *Present* in three categories: correct recognitions of *Present* Mentioned items, incorrect (false) recognitions of *Present* Negated items, and incorrect (false) recognitions of *Present* Not Mentioned items. While ostensibly the comparison was between raw numbers of *Present* answers, they were a measure of correct recall of Mentioned items and a measure of false recall of Negated and Not Mentioned items.

### Procedure

The experiment was conducted in a computer lab. Participants attended the experiment in groups of 6 to 10. Each individual was randomly assigned to one of two conditions (*explicit negation* vs. *implicit negation*) and one version of the recording. Participants were instructed to carefully listen to the recording and to write down a nickname and the filename (number) of the recording they were assigned to (in order to match the version of the recording with the answer sheet). Subsequently, participants were given a filler task (unrelated to the recording) which lasted about 5 minutes. In the Short Delay treatment, participants were then given the memory test. Individuals in the Long Delay condition were released after the filler task, not informed that the experiment would continue at a later date. In this condition, participants received the memory test one week later, on short notice. All participants were also asked to sign the tests with their nickname and the recording number, so that we could match the version of the description with the memory test.

### Experimental design

The design included two between-subject factors: type of negation (Explicit/Implicit) and delay (5 minutes/1 week). Moreover, there was a within-subject factor: the type of information (items being mentioned, negated or not mentioned). Therefore, a mixed counterbalanced 2x2x3 design was used.

## Results

### Implicit vs explicit negation and false recall of negated items

Analyses were conducted using ANOVA. The most important result was that Type of Negation did not significantly influence the number of Negated items (incorrectly) classified as ‘Present’ (*M* = 1.96, *SD* = .12 for Implicit Negation and *M* = 1.90, *SD* = .13 for Explicit Negation, *F*(1,164) = .124; *p* = .726; *η*^*2*^_*p*_ = .001). As this finding is a nonsignificant effect, Bayesian probabilities for the null and alternative hypothesis were calculated (based on [24]), yielding *p(H0|D)* = .924 and a *p(H1|D)* = .076, meaning that the Bayesian probability that Implicit vs Explicit Negation influences the number of ‘Present’ answers to Negated items was 7.6%. This effect was neither significant in the 5 minute delay treatment (*M* = 1.05, *SD* = .18 for Implicit Negation, *M* = .88, *SD* = .18 for Explicit Negation; *p* = .497; *η*^*2*^_*p*_ = .003; *p(H0|D)* = .873, *p(H1|D)* = .127) nor in the one week delay treatment (*M* = 2.88, *SD* = .17 for Implicit Negation, *M* = 2.93, *SD* = .18 for Explicit Negation; *p* = .839; *η*^*2*^_*p*_ < .001; *p(H0|D)* = .902, *p(H1|D)* = .098).

### Negation-related false memories (NRFM)

Analyses were conducted using within-subjects GLM, all pairwise comparisons calculated with Bonferroni correction. The type of information regarding items (Mentioned, Negated or Not Mentioned) significantly influenced the number of items classified as ‘Present’ in the recording (*F*(2,334) = 124.982; *p* < .001; *η*^*2*^_*p*_ = .428). The number of (correct) ‘Present’ answers to Mentioned items was the highest (*M* = 4.20; *SD* = 1.34) and significantly higher than the number of (incorrect) ‘Present’ answers to Negated and Not Mentioned items (both *p* < .001). The difference between Negated (*M* = 1.98; *SD* = 1.50) and Not Mentioned (*M* = 1.99; *SD* = 1.39) was nonsignificant (*p* > .99). Pairwise comparisons of expected marginal means for Negated and Not Mentioned items in the 5 minute and one-week delay treatments revealed that the difference in the number of ‘Present’ answers to Negated and Not Mentioned items was significant for Implicit Negation after the 5 minute delay (*M* = 1.05, *SD* = .18 for Negated items, *M* = 1.88, *SD* = .21 for Not Mentioned items; *p* = .003) and for Explicit Negation after the one-week delay (*M* = 2.93, *SD* = .18 for Negated items and *M* = 2.15, *SD* = .21 for Not Mentioned items, *p* = .005).

### Negation effects vs. random chance answers

**Fig 1:**
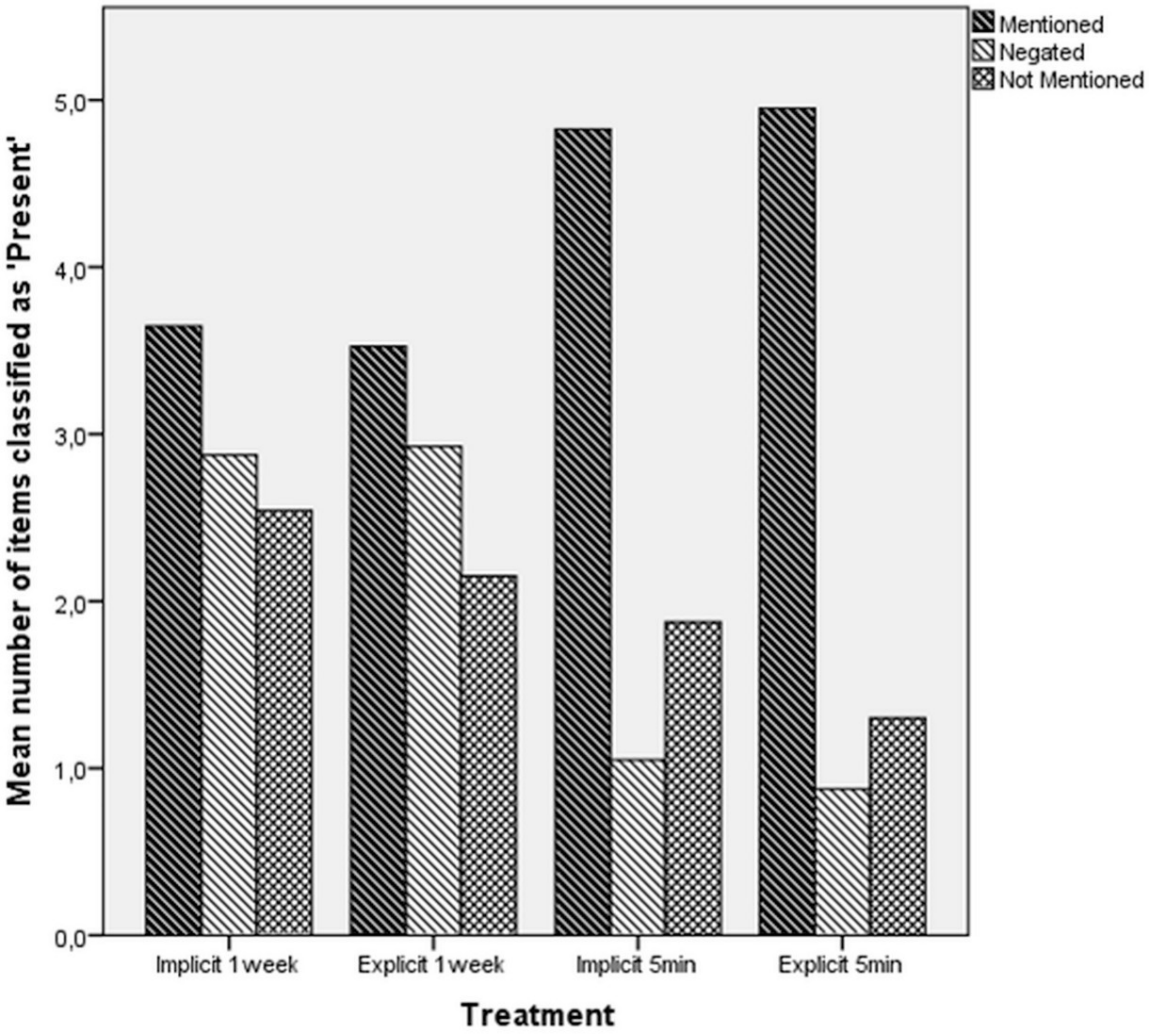
Mean numbers of ‘Present’ answers across information types and treatments.

Analyses were conducted using one-sample t-tests. The number of ‘Present’ answers to Negated items after the one week delay was not significantly different from 50% random chance (*t*(87) = -.803; *p* = .424), while for the 5-minute delay it was significantly lower (*t*(79) = -16.807; *p* < .001). The number of ‘Present’ answers to Not Mentioned items was significantly lower from random chance both after a one week delay (*t*(87) = -4.993, *p* < .001) and after a 5-minute delay (*t*(87) = -8.528, *p* < .001). Means, SDs and 95% CIs for ‘Present’ answers across Type of Information, Negation Type and Delay are presented in Table 1. The mean numbers of ‘Present’ answers across types of information and treatments (delay and negation type) are presented in Fig 1.

**Table 1:** Expected marginal means for ‘Present’ answers across information types and treatments.

Delay	Negation Type	Type of information	M	SD	95% CI	
1 week	Implicit	Mentioned	3.65	1.14	3.309	3.982
		Negated	2.88	1.28	2.548	3.202
		Not Mentioned	2.54	1.15	2.164	2.919
	Explicit	Mentioned	3.53	1.30	3.156	3.894
		Negated	2.93	1.10	2.566	3.284
		Not Mentioned	2.15	1.23	1.736	2.564
5 minutes	Implicit	Mentioned	4.83	1.06	4.456	5.194
		Negated	1.05	1.28	.691	1.409
		Not Mentioned	1.88	1.42	1.461	2.289
	Explicit	Mentioned	4.95	1.22	4.581	5.319
		Negated	.88	.85	.516	1.234
		Not Mentioned	1.30	1.51	.886	1.714

### Impact of negation type on recognition of Not Mentioned items

Further investigation into the numbers of (incorrect) ‘Present’ answers to Negated and Not Mentioned items dependent on Negation Type and Delay demonstrated that in the Implicit Negation treatment, there was a higher number of ‘Present’ answers to Not Mentioned items than in the Explicit Negation group. The difference was statistically significant with Delay excluded: *F*(1, 167) = 5.62; *p* = 0.02; *M* = 2.24, *SD* = 1.31 for Implicit Negation; *M* = 1.73, *SD* = 1.43 for Explicit Negation.

Predictably, the number of ‘Present’ answers to Mentioned items was significantly higher for the 5 minute than for the 1 week delay (*M* = 4.89; *SD* = 1.14 vs *M* = 3.59; *SD* = 1.21; *p* < .001), the number of ‘Present’ answers to Negated items was higher for the 1 week than the 5 minute delay (*M* = 2.90; *SD* = 1.19 vs *M* = .96; *SD* = 1.08; *p* < .001), and the number of ‘Present’ answers to Not mentioned items was higher for the 1 week than the 5 minute delay (*M* = 2.36; *SD* = 1.20 vs *M* = 1.59; *SD* = 1.48; *p* < .001).

## Discussion

The presented study is a continuation of earlier research by Maciuszek & Polczyk [10], in which the NRFM (Negation Related False Memories) effect was shown. NRFM led to a significantly higher number of false alarms regarding negated than not mentioned items (after a one-week delay). This finding raised a question whether the NRFM effect is to be explained by cognitive processing of explicit negation (i.e. negative statements) or whether it can be accounted for within a broader theoretical scope. It is possible that recalling an item mentioned in a given source is easier than recalling whether the item was mentioned within an affirmative or negative statement (especially after a weeklong delay). In other words, it may be easier to recall individual words within statements than the syntactic structure of these statements.

On the one hand, the presented research served as a replication of the NRFM effect using explicit negation. More importantly, however, we tested whether the NRFM effect can be reproduced when negation is expressed in affirmative statements–implicitly. We wanted to investigate the consequences of implicit negation on memory of negated items. Our predictions were based on the assumption that implicit negation is different from explicit negation in the following ways:

1. Implicit negation may require deeper cognitive processing in order to derive meaning, which could cause better memory retention,
2. It may lead to a higher level of cognitive load (in order to derive meaning), which coupled with a time-critical nature of understanding auditory material would be detrimental to memorizing,

The two above effects could coincide, leading to a similar quality of recall for explicit and implicit negation.

Results can be summarized in four key points. Firstly, results of the previous research on NRFM were replicated: with short delay there were no significant differences between the number of falsely recalled negated and not mentioned items. However, after a one-week delay the number of falsely recalled explicitly negated items was significantly higher than the number of falsely recalled not mentioned items. Secondly, there were no differences in false recall between implicit and explicit negation, both after a short and a long delay. There is statistical evidence that not only were the differences insignificant, but the probability that the effects are present is very low. Thirdly, an interesting relation between the type of negation (implicit vs explicit) and recall of not mentioned items was detected. In the Implicit Negation treatment, there was a higher number of falsely recalled not mentioned items than in the Explicit Negation group. After a five-minute delay, the number of falsely recalled not mentioned items was higher for Implicit Negation than for Explicit Negation. After a one-week delay the effect was not significant, but still the number of falsely recalled not mentioned items was higher for Implicit than Explicit negation, leading to NRFM being absent in the Implicit Negation one-week delay treatment (caused by a high number of falsely recalled not mentioned items, rather than a low number of falsely recalled negated items; the number of falsely recalled negated items in the two groups was virtually identical). Finally, the most predictable result (but still worth mentioning) was the influence of delay on false recall: the longer delay led to an increased number of falsely recalled items, both implicitly/explicitly negated and not mentioned. Conversely, the number of correctly recalled mentioned items was lower after the one-week delay than after 5 minutes.

There is very little empirical evidence on the cognitive processing of implicit negation. We are not aware of any research concerning the influence of implicit negation on memory, and particularly comparing the effects of explicit and implicit negation on memory performance. The presented study demonstrated that informing about the absence of items by means of implicit negation yields similar, if not identical results as explicit negation: it led to a similar level of false recall (i.e. recognition of negated items as present in the description).

Kaup & Zwaan [21] used explicit and implicit negation to state the absence of items. Although their research investigated the short-term accessibility of text information rather than memory processes, it provides a comparison of explicit and implicit negation. Participants were presented with narratives, the penultimate sentence of which contained a color term and stated the presence or absence of said color within a context. The absence of the color was stated via a negative (explicit negation) or an affirmative statement (implicit negation)–e.g. *Susan liked the bike*, *she was glad that it did not have a blue frame* (negative/absent) or *Susan liked the bike*, *she only wished that it had a blue frame* (affirmative/absent). The accessibility of the color term was measured by means of a probe-recognition task either 500 ms or 1500 ms after participants had read the sentence mentioning the color term. After a 1500 ms delay, the participants’ reaction time to the probe-recognition task was longer when the color was stated as absent in the description, and shorter when the color was stated as present. It means that after a certain point in the comprehension process, concepts which are present in the state of affairs are more available than concepts absent from this state of affairs. Important for the present study, however, is that the response times were similar for explicit and implicit negation both after a 500ms and a 1500ms delay. It means that the accessibility of negated text information was independent from the type of negation. Additionally, the authors analyzed reading times (i.e. amount of time required to read) of the target sentences. The type of negation (explicit and implicit) did not have any significant influence on reading times. It can be inferred that the affirmative absent (implicit negation) was similarly difficult to process as the negative absent (explicit negation) condition.

The above study concerned the short-time impact of negation and immediate, transient representations based on it. Our study addresses the long-term impact of negation on memory performance. Neither of these studies showed a difference between explicit and implicit negation. While the processing of implicit and explicit negation in the study by Kaup & Zwaan [21] was similar, it does not necessarily mean that the nearly identical number of falsely recalled for explicit and implicit negation presented in our study is caused by an identical memory mechanism. Indeed, results do not seem consistent with this explanation, due to the difference in the number of Not Mentioned items incorrectly recognized as Present in the explicit and implicit negation treatments. Were the memory mechanisms identical for these two types of negation, such a difference would have no basis to occur. It seems that the impact of implicit and explicit negation on memory processing may be different in at least two potential ways: implicit negation may lead to a deeper level of processing (i.e. inferring the absence of an item), and a higher cognitive load. These two factors might counteract one another, leading to a similar level of false recognition in both types of negation.

The assumption that implicit negation leads to a higher cognitive load is based on the fact that in the present study the negative sentences were relatively simple, having one negation at most (e.g. *There is no fireplace in the living room*). Explicit negation is a direct, literal information about the absence of an item, while implicit negation indirectly states the absence, requiring inference. The presented study utilized an audio recording, the processing of which is time-sensitive. In most research on negation, the information is presented as text and reading time is a measure of the text’s difficulty. The reading time is longer for negated sentences. An audio recording makes participants unable to control the speed at which information is fed, in order to adjust to the difficulty of said information (whether it consists of affirmative or negative statements, as well as whether it utilizes implicit or explicit negation). Should a given statement require more time to process than the recording provides, participants are forced to begin processing the next statement while still engaged in processing the previous one. This requires the participants to either stop processing the previous sentence before it is fully understood, to delay processing the next sentence (eventually forcing them to skip content as they are engaged in processing a previous sentence), or to process more than one sentence simultaneously with limited cognitive capabilities. Let us assume that this is more common for implicit negation. Participants who were forced to process the sentences faster than they were able to would have a worse recall of the whole description, as well as reduced trust in their memory of the description (e.g. [25]). This would make them more prone to guessing whether items not mentioned in the description may or may not have gone by unnoticed while they were processing another sentence. In other words, they simply would not be sure what they may have missed or forgotten. This would lead to an increased number of ‘Present’ answers to Not Mentioned items for Implicit Negation than for Explicit Negation–a conclusion consistent with our results. However, the present study does not provide direct empirical evidence to verify such an explanation, which is to be tested in future research.

The presented study confirmed the expectation that absence of items stated within affirmative sentences (implicit negation) may generate a similar level of false recall as direct statements of absence within negative sentences (explicit negation). The effect described in Maciuszek & Polczyk [10] which inspired the present study–a significantly greater number of falsely recalled negated than not mentioned items after a weeklong delay–may be explained by it being easier to recall objects mentioned within a sentence than to remember whether said objects were affirmed or negated. For explicit negation, this effect may be reinforced by how negation is processed. Research on negation shows that negative sentences are more difficult to process than affirmative sentences. Moreover, the early steps of cognitive processing cause an activation of the negated state of affairs (see [26]). These mechanisms lead to the following consequences for memory: (1) Affirmative sentences are remembered better than negative sentences, and (2) a transient representation of the negated objects can activate during the memory test, leading to false recall. Moreover, memory errors often take the form of converting negative sentences into affirmatives (e.g. *Jim is not responsible* might be remembered as *Jim is responsible*—see [9], see also [27]. These types of errors are particularly typical for uni-polar descriptions—a kind of description which has no well-defined opposite construct / antonym (e.g. *not charismatic* does not clearly suggest any particular trait). A bi-polar description has a clear opposite construct which is easily accessible (e.g. *Tom is not guilty* would be encoded as *Tom is innocent*) [9]. Negation in our study concerns descriptions which are uni-polar, that is, where negation does not point to a clear alternative (e.g. *There was no fridge*). In this case *dissociation errors* (i.e. loss of the negation marker, resulting in negatives turning into affirmatives) are likely. The present research suggests that such errors and mechanisms of cognitive processing are not specific for negative statements and explicit negation, but may be elicited using implicit negation as well. For example, the statement *John wishes he were happy* may be remembered as J*ohn is happy* just as easily as the statement *John is not happy*, though both sentences (bi-polar) state that John is indeed unhappy.

The contemporary contextual approach focuses on the importance of context on processing negation [28,26], stating that whether processing negation is easier or more difficult depends on the context in which negation is presented. It seems this assumption may also be relevant to implicit negation. Future research should investigate whether context factors moderate implicit negation processing and memory. Research on implicit and explicit negation may provide important implications for theories of memory and language processing, and therefore investigating the underlying mechanisms of understanding and remembering on implicit negation seems worthwhile.

## Supporting information

S1 Dataset Raw data obtained in the presented research. (SAV)
